# Deep learning and metaheuristic optimization for predicting drug release from nanocarrier systems: a data-driven framework for formulation development

**DOI:** 10.3389/fmed.2026.1962360

**Published:** 2026-10-01

**Authors:** Hadil Faris Alotaibi, Saad S. Alqahtani

**Affiliations:** 1 Department of Pharmaceutical Sciences, College of Pharmacy, Princess Nourah Bint AbdulRahman University, Riyadh, Saudi Arabia; 2 Department of Clinical Pharmacy, College of Pharmacy, King Khalid University, Abha, Saudi Arabia

**Keywords:** drug release kinetics, metaheuristic optimization, nanocarrier systems, physicochemical descriptors, sustained-release nanomedicine

## Abstract

Drug-release profiles from nanocarrier systems are governed by nonlinear and multiphasic physicochemical interactions that are difficult to predict using conventional kinetic models. This study presents a data-driven predictive framework that integrates deep neural networks (DNNs) with three bio-inspired metaheuristic optimization algorithms: the Transit Search Optimization Algorithm (TSA), Crocodile Hunting Strategy (CHS), and Osprey Optimization Algorithm (OOA). The models were developed and evaluated using a dataset of 403 nanocarrier formulations compiled from 237 peer-reviewed studies. Predictive performance was assessed using RMSE, MSE, R^2^, Nash-Sutcliffe Efficiency (NSE), and GR125, followed by multi-criteria ranking with TOPSIS and statistical validation using the Kruskal–Wallis and Friedman tests. Among the evaluated models, the OOA-optimized DNN achieved the best overall performance, reaching a minimum RMSE of approximately 1.01 and an R^2^ of approximately 0.99 while exhibiting stable convergence and statistically significant improvements over the competing optimization strategies. The proposed framework provides a robust in-silico tool for modeling complex drug-release behavior, reducing experimental screening requirements, and supporting data-driven formulation optimization, Quality by Design (QbD), and Model-Informed Drug Development (MIDD) for nanocarrier systems.

## 1 Introduction

Physiological barriers and low drug bioavailability are the main factors that cause most of the effective treatments to lose their power, especially treatments for diseases that directly affect the central nervous system (CNS). The blood-brain barrier (BBB) is probably the most limiting of the human body interfaces among these barriers as it prevents almost 90% of therapeutic agents from reaching their target sites (1, 2). The number of standard drugs that can cross the BBB is less than 1%, this is mainly due to the fact that the BBB is very selective and has tightly regulated molecular transport mechanisms. These constraints have motivated the development of advanced drug delivery strategies capable of improving transport efficiency and therapeutic outcomes (3). Nanocarrier-based drug delivery systems have become an appealing solution to the problem of drug delivery across the BBB (4, 5). In this way, drugs can be protected from premature degradation, their stability can be raised, and targeted delivery can be enhanced. Different types of nanocarriers, such as polymeric nanoparticles, lipid-based systems, and inorganic nanostructures, have been developed with modified physicochemical properties for better interaction with cells and higher permeability. The characteristics of the particle, such as size, surface charge, morphology, encapsulation efficiency, and surface functionalization, play a major role in biological distribution and therapeutic performance (6, 7). Besides that, drug release from nano-sized carriers is an incredibly important clinical outcome factor, other than just providing the target site. The release profile is what essentially determines the bioavailability, the duration of the action, and the dosing frequency, and it is affected by quite complicated mechanisms such as diffusion, polymer degradation, erosion, and matrix swelling (8, 9). However, the degree of nonlinearity and system variability may differ depending on formulation design and experimental conditions.

Traditional mathematical models such as Higuchi, Korsmeyer-Peppas, and first- or zero-order kinetics have been used broadly to explain drug release behavior. Nevertheless, these models frequently depend on simplification assumptions like a homogeneous matrix composition and constant diffusion coefficients, which are almost never the case in real systems (10). Moreover, differences in experimental setups and incomplete characterization of nanoparticles in various studies result in additional variations, thereby making the prediction of drug release less accurate when conventional analytical methods are employed. Considering controlled release as a determining factor of nanocarrier-mediated delivery across physiological barriers like the BBB, the demand for predictive tools capable of modelling complex, non-linear release behaviour is increasing. Currently, research findings have been using machine learning for drug release prediction (11, 12). Therefore, more flexible and data-driven modeling approaches are increasingly being explored to complement classical kinetic descriptions. In this context, there is growing interest in exploring more advanced learning architectures capable of capturing nonlinear relationships in heterogeneous datasets.

Recent attempts to computationally model drug release are mostly divided into three categories: machine learning-based prediction, AI-assisted specialized delivery systems, and physics-driven simulation approaches. Data-driven modelling advancements leading to the use of machine learning as a tool for predicting drug release have been substantiated by various types of formulations. In most papers, classical regression and tree-based models have been utilized to estimate dissolution or kinetic results based on the formulation inputs with noticeable predictive performance. As an illustration, random forest and gradient boosting techniques were applied to regenerate tablet-based dissolution profiles (13), whereas multivariate regression methods like PLS have been studied for release timing in targeted delivery systems (14).

These works collectively demonstrate that ML-based release prediction is becoming more achievable; however, present attempts are still mostly limited to conventional dosage forms and mostly use traditional learning architectures instead of deep neural networks. The second research line has concentrated on the usage of AI in specialized and application-specific delivery platforms. Machine learning-based models have been implemented in personalized delivery scenarios to predict drug loading and release kinetics (15), and to characterize pH-responsive behaviour in liver-targeted nanocomposites with high accuracy (16). The same approaches have been taken for microneedle and transdermal systems, where ensemble models and deep networks were employed to predict permeation or release properties and thus, assist formulation development (17, 18). These studies highlight AI's potential to improve performance prediction in various delivery technologies that are just emerging. However, their scope is still limited to specific cases, usually a single carrier type, and they do not consider wide physicochemical descriptors or generalized modeling frameworks. In addition to data-driven ML methods, there are a few studies that used physics-based and computational simulation techniques to explore drug release mechanisms. Monte Carlo simulations have been employed to study how the influence of lattice geometry and structural features on diffusion-driven release changes (19), thus providing mechanistic insight but requiring system-specific assumptions that limit their generalizability. There have also been proposals for optimization-driven formulations to create release profiles under uncertainty using dissolution-based inverse models, and model-informed formulation strategies have been used to facilitate sustained-release development via PBPK and virtual evaluation pipelines (20).

These pioneering mechanistic and design approaches, while valuable, also do not provide a solution for data-integrated prediction that spans heterogeneous nanocarrier systems. However a significant gap in the literature remains despite these innovations. Most of the existing studies have been mainly focused on tablets, patches, microneedles, or isolated nanocomposite systems; they use conventional ML models and usually predict only one outcome without considering multidimensional release behavior. Importantly, limited studies have explored the integration of physicochemical descriptors with deep learning. This study aims to extend existing approaches by integrating by creating a DNN-based framework that is optimized by nature-inspired algorithms and assessed by k-fold cross-validation, TOPSIS-based performance ranking, and Kruskal–Wallis and Friedman tests for statistical robustness (Figure 1). The primary objective of this work is to construct a predictive model that can simulate the nonlinear drug release behavior of nanocarrier systems on the basis of the comprehensive physicochemical descriptors. Three nature-inspired algorithms, the Transit Search Optimization Algorithm (TSA), Crocodiles Hunting Strategy Optimization (CHS), and Osprey Optimization Algorithm (OOA), are utilized to optimize a deep neural network (DNN), and k-fold cross-validation, Kruskal–Wallis and Friedman testing, and TOPSIS-based multi-metric ranking are employed for the performance assessment of the DNN. The TSO, CHS, and OOA are employed in this study as hyperparameter optimization strategies for the DNN. These algorithms are not intended for mechanistic interpretation but are used to enhance predictive performance by optimizing model parameters in a high-dimensional and nonlinear search space. The selection of multiple metaheuristic algorithms is motivated by their complementary search behaviors, including exploration and exploitation balance, convergence characteristics, and robustness in solving complex optimization problems. Including multiple optimizers enables a fair comparative assessment of optimization strategies under identical modeling conditions. It is emphasized that the interpretability of the framework is derived from the input physicochemical descriptors and their relationship to predicted release behavior, while the optimization algorithms serve only as performance enhancement tools rather than explanatory models.

**Figure 1:**
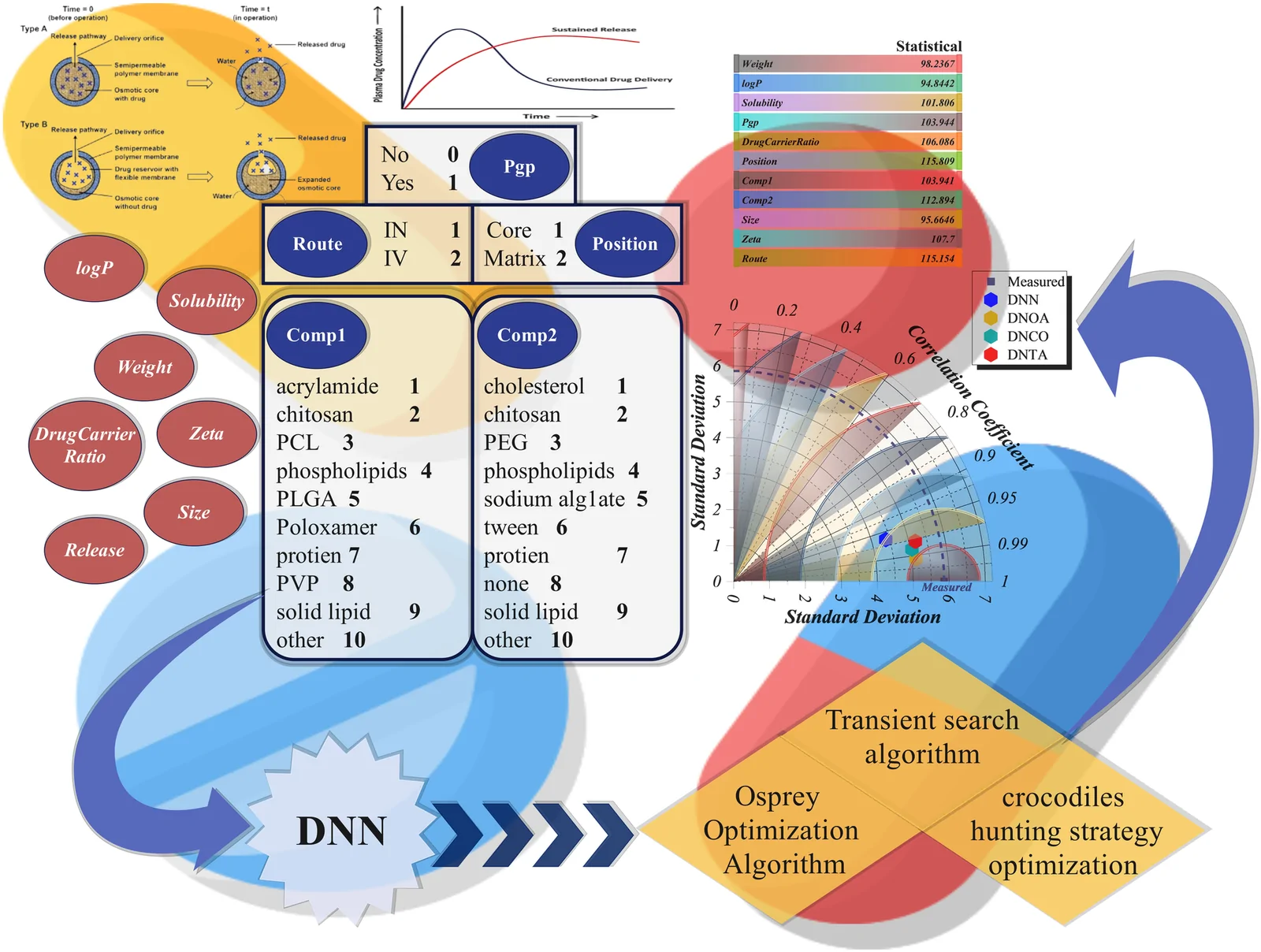
Integrated framework illustrating how physicochemical nanoparticle descriptors, drug-release mechanisms, deep neural network modeling, and advanced metaheuristic optimizers collectively support the prediction and optimization of drug-release behavior from nanocarrier systems.

The goal of this study is to provide a robust and comparative predictive framework rather than to replace mechanistic models, but to complement them in understanding complex drug release behavior.

The present study focuses exclusively on *in vitro* drug release behavior from nanocarrier systems using formulation- and carrier-related physicochemical descriptors. The developed predictive framework is designed to model experimentally reported release profiles under *in vitro* conditions and does not extend to the prediction of *in vivo* pharmacokinetics, bioavailability, or systemic drug disposition processes such as carrier clearance, plasma protein binding, or tissue distribution. While such physiological processes critically influence *in vivo* drug exposure, they are not represented in the available *in vitro* release datasets and therefore are not included in the current modeling framework.

## 2 Dataset and preprocessing methods

A systematic literature-mining strategy was carried out to build a manually curated, multisource dataset focusing on nanoparticle delivery from the nose to the brain. Strict inclusion and exclusion criteria were used to ensure reproducibility and scientific rigor, resulting in 237 peer-reviewed studies that met all the requirements (21). There are a few existing datasets that combine formulation attributes, biological behavior, and pharmacokinetic results for comparative nasal vs. intravenous delivery, thus positioning this dataset as the main contribution of the study. The final design matrix consisted of 403 records and 24 variables that included both discrete and continuous descriptors. The features related to the formulations were particle size, zeta potential, encapsulation efficiency, drug localization (core/shell), shape, and surface modifications, together with exposure time and *in vivo* accumulation. Three normalized pharmacokinetic ratios were determined as primary output variables to limit inter-study variability: $Y_{1}=\frac{\mathrm{AU}C_{\mathrm{brain}}}{\mathrm{AU}C_{\mathrm{plasma}}}$, $Y_{2}=\frac{C_{\max,\mathrm{brain}}}{C_{\max,\mathrm{plasma}}}$, and $Y_{3}=\frac{T_{\max,\mathrm{brain}}}{T_{\max,\mathrm{plasma}}}$. These ratio-based targets made it possible to have a standardized evaluation of the targeting efficiency without being affected by the absolute dosing differences.

Preprocessing was done to make sure the analysis was consistent, and there was no bias in the statistics. Records with incomplete pharmacokinetic information were removed so that the data integrity is kept, while the remaining missing values were filled in by median (continuous) and mode (categorical) methods because these are more robust against skewed distributions. Min-Max normalization was used to remove scale dominance from the different physicochemical variables, and categorical descriptors were one-hot encoded to prevent the model from learning unintended ordinal relationships. Outliers were checked and fixed to lessen the distortion of model training. Initial statistical investigation revealed that there were no strong linear dependencies as per Pearson correlation analysis, and Principal Component Analysis also showed a variance structure that was spread across features. The results here pointed to the dataset having high dimensionality and nonlinear behaviour, which is why the use of sophisticated deep learning architectures and nature-inspired optimization instead of conventional parametric modelling was justified.

### 2.1 Distributional diversity and nonlinear feature interactions in nanocarrier physicochemical descriptors

The scatter matrix in Figure 2 illustrates considerable differences in the physicochemical descriptors, with several variables having non-Gaussian, skewed, or widely spread distributions. This variation is a direct consequence of the different formulation strategies that have been employed in the various nanocarrier studies from which data were pooled. The lack of obvious linear clustering in most pairwise comparisons also suggests that simple or direct correlations cannot explain drug release. The scatter diagrams show that there are nonlinear and multivariate interactions among the formulation parameters. The statements made here are the reasons which advanced nonlinear modeling is required, and thus the methodological departure from earlier studies that were mainly linear or heuristic release predictions is reinforced.

**Figure 2:**
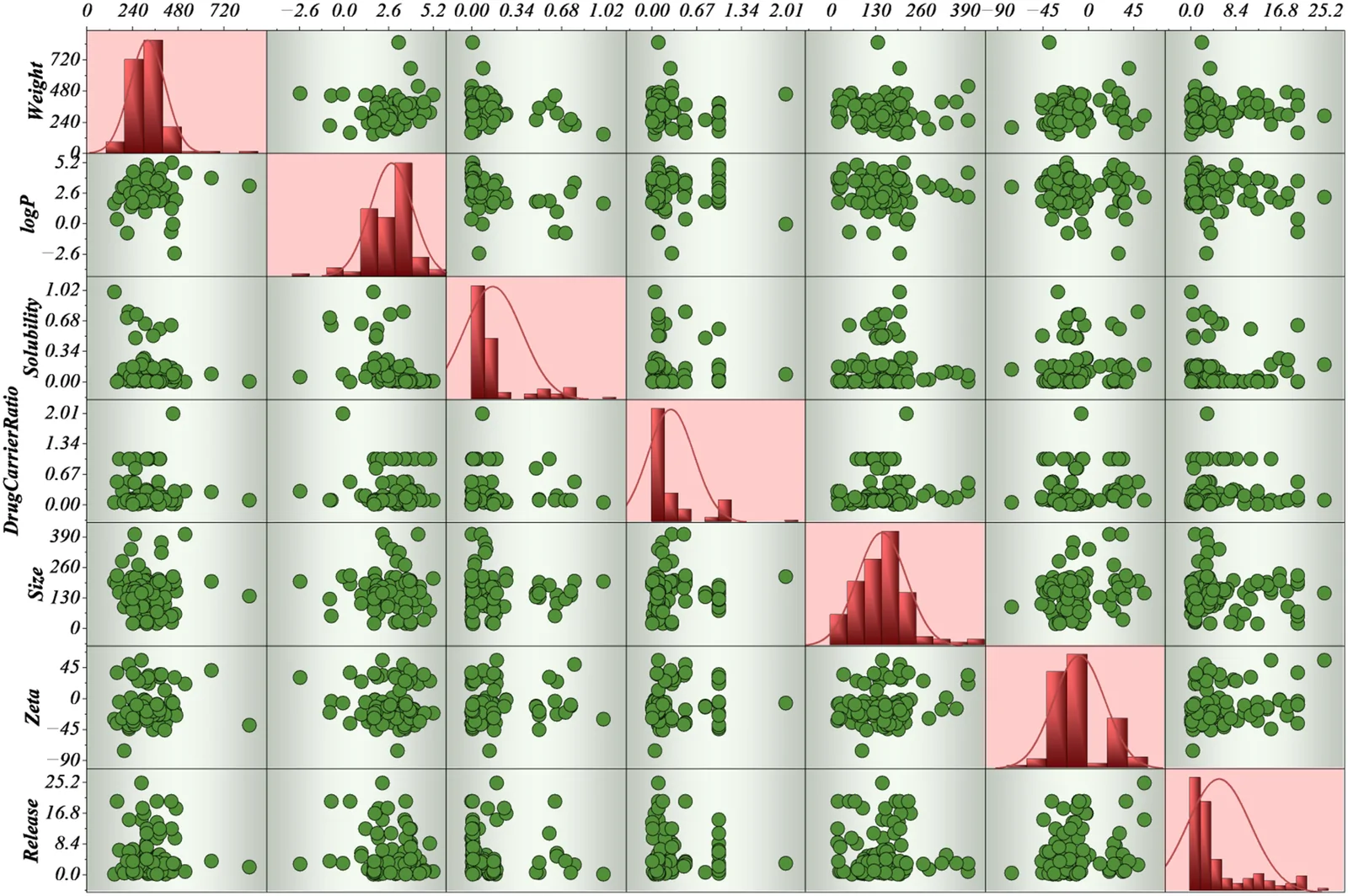
Scatter matrix plot for the normal features and the target variable, providing a general overview of their pairwise relationships, distribution patterns, and potential interdependencies.

### 2.2 Descriptive statistics revealing non-Gaussian and heterogeneous feature distributions across nanocarrier formulations

Table 1 presents an overview of the statistical profiles of the dataset that have significantly diverse and non-Gaussian feature spaces. This confirms that the parameters for the formulation vary considerably in different studies. Some of the continuous variables have a wide numerical range, pronounced dispersion, and elevated kurtosis. These indicate that distributions are asymmetric and heavy-tailed rather than symmetrically patterned, as would be expected for idealized physicochemical systems. Such variability is due to the diversity of the nanoparticle platforms, which cover differences in particle size, charge, solubility behavior, and drug-to-carrier ratios. At the same time, the presence of uniformly distributed discrete variables further increases the structural complexity of the dataset. Principal figures of Size, Zeta potential, Drug-Carrier Ratio, and Release have strong non-normal characteristics, which means that their relationships with drug-release dynamics are not likely to be of simple linear types. The combination of skewed distributions, high variance, and mixed categorical–continuous representations suggests that the underlying interactions between formulation parameters are multidimensional and nonlinear, which matches the patterns seen in the scatter-matrix analysis. These statistical features point to the restrictions of conventional linear or heuristic modeling methods and thus warrant the use of sophisticated nonlinear learning frameworks. Deep neural networks, which are supported by strong optimization algorithms, are therefore in a good position to capture the underlying release mechanisms that conventional analytical models fail to represent, by operating within a feature space characterized by heavy-tailed distributions and complex interdependencies.

**Table 1:** Evaluation of input variables and the target variable for robust model development.

Distribution	Variable	Indicator					
		Lower bound		Upper bound		Mode	
Uniform	Pgp	0		1		1	
	Position	1		2		1	
	Comp1	1		10		5	
	Comp2	1		10		8	
	Route	1		2		1	

		Min.	Max.	Mean	Median	Kurtosis	St. Dev.
Normal	Weight	146.14	853.91	319.05	315.03	8.038	92.62
	logP	−2.56	5.21	2.769	2.93	2.525	1.234
	Solubility	0	1	0.159	0.071	2.550	0.220
	DrugCarrierRatio	0.007	2	0.285	0.1	4.429	0.343
	Size	19	400	147.69	150.2	1.989	71.46
	Zeta	−76	55.47	−8.941	−15.78	0.098	25.281
	Release	0.1263	25	5.277	3.13	1.143	5.837

To facilitate interpretation of the predictive targets, the pharmacokinetic ratios included in the dataset are explicitly defined and described. These ratios were extracted from the original studies and incorporated as quantitative indicators related to drug release and formulation performance. The selected ratios provide normalized measures that enable comparison across studies with different experimental conditions and formulation characteristics. In addition to reducing scale-related variability, ratio-based descriptors may capture relative changes in drug availability, release behavior, or distribution patterns that are not fully reflected by absolute measurements alone. Although these ratios are associated with pharmacokinetic behavior, they are used in the present study as reported outcome variables within the collected dataset and should not be interpreted as direct measures of *in vivo* bioavailability or systemic drug disposition. Their inclusion is intended to support comparative analysis of formulation-dependent release characteristics across heterogeneous nanocarrier systems.

### 2.3 Non-parametric dual-criterion ranking of key formulation variables using dispersion metrics and Kruskal-Wallis significance

Figure 3 describes a dual-criterion, non-parametric evaluation framework that fuses statistical dispersion-based ranking with Kruskal–Wallis significance testing to locate the most influential features in a heterogeneous drug-release dataset. The integration of rank-based variability measures with group-wise differentiation allows the portrayal to go beyond a standard Sankey plot and offer a methodologically sound manner to depict feature contributions in non-normal and unevenly distributed experimental conditions. The set of formulation and biological descriptors, which always hover around the high-influence/low-*p*-value region of the dual-criterion space, is the one that most probably carries the strongest discriminative capacity. Illustrated examples of such variables are the drug–carrier ratio, zeta potential, and positional/compound-related descriptors, which show both wide statistical dispersion and significant non-parametric separation, while for some other physicochemical attributes, the mid- to low-impact ranges are their clusters. Where there is a combination of high dispersion and significant rank-based differentiation, the nonlinear and multi-factor dependency structure becomes visible, thus providing a data-driven rationale for using deep neural networks and nature-inspired optimizers.

**Figure 3:**
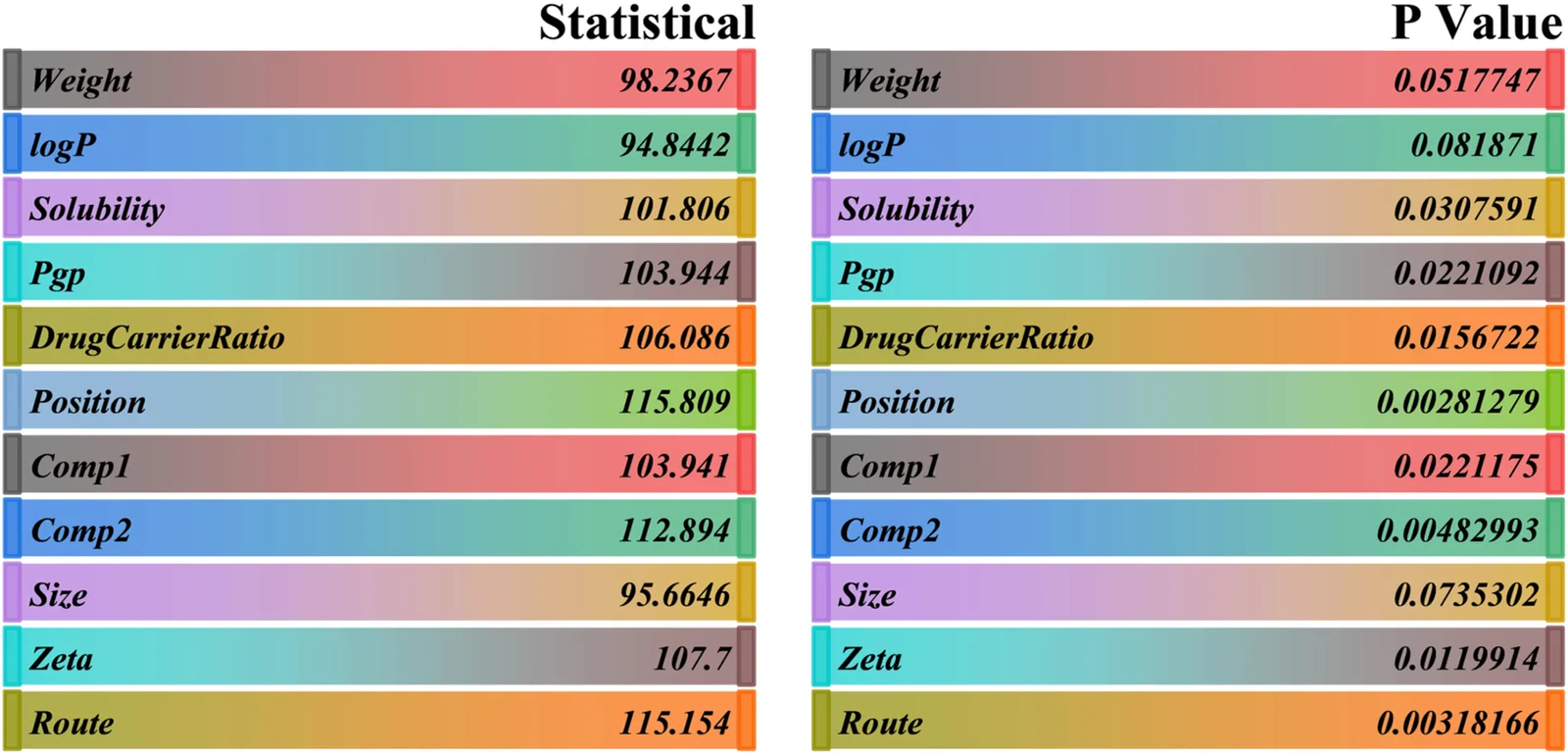
Sankey plot for visualizing the distribution of features in the Kruskal test, for feature selection.

### 2.4 Cross-validation performance of the baseline DNN model for predicting nanocarrier drug-release behavior

To prevent data leakage and ensure independence between training and validation sets, a study-wise grouped k-fold cross-validation strategy is employed. In this approach, all samples originating from the same published study are assigned exclusively to either the training or validation fold, ensuring that no data from a single study appears in both sets. This grouping strategy is essential in heterogeneous literature-derived datasets, where multiple formulations reported within the same study may share experimental conditions, measurement protocols, or underlying system characteristics. Maintaining study-level separation improves the reliability of performance estimation and reduces optimistic bias in model evaluation. Although external validation using an independent dataset would further strengthen generalizability, such a dataset with consistent physicochemical descriptors and standardized reporting was not available. Therefore, model robustness is evaluated using repeated grouped k-fold cross-validation combined with statistical significance testing.

Table 2 presents the cross-validation performance of the baseline DNN model and serves as a first point of reference to the model's predictive power in drug release modeling from nanocarrier systems. The model keeps the RMSE values quite low and the R^2^ scores high in all five folds, with only slight variations from one fold to another. Such stability is proof of the DNN's commendable generalization capacity, notwithstanding the inherent variability of nanoparticle datasets that are influenced by factors like size, surface charge, encapsulation efficiency, morphology, and surface modifications. It is worth noting that the R^2^ values are consistently high, which means that the DNN is able to capture the nonlinear and multi-phasic nature of drug release kinetics that underlie the data much better than mechanistic models of a traditional type. In general, the results are an indication that the baseline DNN is a sound starting point for the study and thus its further improvement by sophisticated optimization approaches such as TSOSA (the CHS, and the OOA) is warranted.

**Table 2:** Outcomes obtained from the implemented K-fold procedure.

Model	Indicator	Number of K-folds				
		K1	K2	K3	K4	K5
DNN	*RMSE*	2.158	2.679	2.496	2.132	2.395
	*R^2^*	0.922	0.882	0.901	0.930	0.914

## 3 Methodology

### 3.1 Overall framework

A complete systematic framework was built to model the complex nonlinear relationship between nanoparticle features and nose-to-brain pharmacokinetic variables. The workflow included creating a dataset by mining multiple sources of literature, performing standardized preprocessing, and designing a DNN for multi-output regression. Three nature-inspired optimization algorithms, TSA, CHS, and OOA, were used to improve the model performance by automatically adjusting the hyperparameters in a given search space. The assessment was done through k-fold cross-validation on various performance metrics, followed by non-parametric statistical testing and multi-criteria ranking using TOPSIS to confirm the accurate and unbiased comparative evaluation.

### 3.2 Deep neural network (DNN)

An end-to-end DNN with multiple outputs was formulated to represent the nonlinear relationship between 24 input descriptors and the three pharmacokinetic ratios. The network layers were sequential, with each utilizing the ReLU activation function to make the optimization process more stable and to avoid the vanishing gradient effect. The set of learnable parameters *θ* (weights and biases) was updated by minimizing a supervised loss function over all outputs (22). To restrain overfitting and thus improve the model's generalization capacity, a number of regularization methods, such as dropout and early-stopping criteria, were used. The hyperparameters that were eligible for optimization included the number of hidden layers, the number of neurons in each layer, learning rate, batch size, activation, and regularization strength, all of which were adjusted automatically rather than manually (23, 24).

### 3.3 Optimization strategy

In order not to depend on heuristic or manually select hyperparameters, a tri-optimizer strategy was utilized that made it possible comparative high-dimensional search under one and the same shared objective function and identical evaluation protocol.

#### 3.3.1 Transit search algorithm (TSA)

TSA was used to globally search the space by successively changing candidate solutions with the help of transit-inspired selection and luminosity-based evaluation (25). Here, TSA balanced broad exploration during early iterations and targeted refinement during the later stages in order to optimize DNN hyperparameters, thus enhancing the convergence stability in nonlinear landscapes.

#### 3.3.2 Crocodile hunting strategy (CHS)

CHS has been employed as a local search method to gain higher local search refinement. The algorithm, through the chaser and ambusher stages, intensifies the exploitation around the most promising regions of the hyperparameter space (26). In the system of equations, CHS assisted in the precise adjustment of the learning rate and network width, thereby decreasing the predictive error variance over different validation folds.

#### 3.3.3 Osprey optimization algorithm (OOA)

Third optimizer OOA was linked with the proposal to keep the solution pool varied and lengthen the overall search. The two-stage update method (exploration of the area by shifting the position and exploitation by controlled adjustment) allowed the search to adapt to high-dimensional parameter tuning (27, 28). OOA was instrumental in finding those configurations that provided consistent performance for all three target outputs.

### 3.4 Model validation and statistical analysis

To fairly evaluate the generalization ability of the models, all the optimally trained models were subjected to k-fold cross-validation and standard regression metrics, such as Mean Squared Error (MSE), Nash–Sutcliffe Efficiency (NSE), Gradient Ratio within ±25% (GR125), Root Mean Squared Error (RMSE), Mean Absolute Error (MAE), and the Coefficient of Determination (R^2^), were calculated. Non-parametric Kruskal–Wallis and Friedman tests were used to detect statistically significant differences in the performance of the three optimization methods to avoid normality assumptions. Lastly, a Technique for Order Preference by Similarity to Ideal Solution (TOPSIS) framework was implemented to merge the multi-metric results into one single ranking, thus facilitating the most robust model configuration's objective and reproducible selection.

#### 3.4.1 Performance evaluators strategy

*RMSE* and *MSE* served as measures to indicate the absolute prediction error, where RMSE was the scale-sensitive measure of the deviation between the observed and predicted ratios, while MSE was a way of model optimization by providing a stable and smooth objective function during training. These measures, in concert, indicate the extent and the distribution of residual errors in the dataset. The coefficient of determination *(R^2^)* was used to measure the proportion of variance of the target outputs that the model was able to explain and, thus, was an indicator of the model's ability to learn the underlying trends related to the release instead of just minimizing the error magnitude. Nash-Sutcliffe Efficiency *(NSE)* was a measure of the model's predictive performance relative to the observed data's mean and, hence, was very relevant for ratio-based biological responses where both the systematic and random deviations have to be accounted for. *GR125* was an indicator of operational trustworthiness by telling the fraction of the predictions that were within ±25% of the experimental values and, thus, was a threshold-based measure easily interpretable that was in line with the real-world acceptability ranges. Equations 1–5 presents mathematical approach for evaluators used in the study.

$$\mathrm{RMSE}=\sqrt{\frac{1}{n}\overset{n}{\underset{i=1}{\sum }}\;{\left(y_{i}-{\hat{y}}_{i}\right)}^{2}}$$ (1)

$$\mathrm{MSE}=\frac{1}{n}\overset{n}{\underset{i=1}{\sum }}\;(y_{i}-{\hat{y}}_{i}{)}^{2}$$ (2)

$$\$R^{2}=1-\frac{\sum_{i=1}^{n}\;{\left(y_{i}-{\hat{y}}_{i}\right)}^{2}}{\sum_{i=1}^{n}\;{(y_{i}-\overset{)}{y}}^{2}}\$$$ (3)

$$\$\mathrm{NSE}=1-\frac{\sum_{i=1}^{n}\;{\left(y_{i}-{\hat{y}}_{i}\right)}^{2}}{\sum_{i=1}^{n}\;{(y_{i}-\overset{)}{y}}^{2}}\$$$ (4)

$$\mathrm{GR}125=\frac{1}{n}\overset{n}{\underset{i=1}{\sum }}\;I\left(\;\frac{y_{i}-{\hat{y}}_{i}}{y_{i}}\leq 0.25\right)\times 100$$ (5)

### 3.5 Methodological transparency and contribution clarity

To ensure reproducibility and methodological transparency, detailed descriptions of dataset construction, feature selection, data preprocessing, model architecture, and hyperparameter optimization procedures have been provided. The deep neural network structure and the implementation of nature-inspired optimization algorithms are explicitly described to allow replication of the proposed framework. The proposed model is designed as a data-driven predictive tool for *in vitro* drug release behavior across heterogeneous nanocarrier formulations, rather than a universal predictive system applicable to all drug delivery contexts. Accordingly, claims regarding generalizability have been revised to reflect performance within the studied dataset rather than across all possible nanocarrier systems. The contribution of this study lies in the integration of physicochemical descriptors with optimized deep learning and multi-criteria evaluation techniques, providing a comparative framework for analyzing formulation-dependent release behavior under *in vitro* conditions.

## 4 Result & discussion

### 4.1 Hyperparameter optimization and convergence characteristics of DNN models tuned by TSA, CHS, and OOA algorithms

Table 3 displays the optimized DNN hyperparameters and the convergence metrics that were derived by applying the TSA, the CHS, and the OOA for 200 iterations. The three metaheuristics show clear distinctions in performance. OOA exhibited the most robust optimization behavior as it reached the lowest minimum RMSE (1.011), the lowest mean RMSE (1.350), and the smallest range between minimum and maximum error values, thus indicating the best convergence stability. The less pronounced oscillation and steeper decline of the error curve indicate that there was more effective exploitation during the late-stage refinement. In all the multi-criteria evaluations, CHS always came second, hence providing moderate improvements but showing a slower and more irregular convergence trajectory. The weakest optimization capability of TSA is illustrated by higher error levels and delayed convergence, which are indicative of limited parameter refinement toward the optimum. The hyperparameter trends also unveiled the different search tactics that the optimizers adopted. The OOA settled for a deeper network (128-128-64) with a reasonable learning rate, a configuration that makes sense considering the nonlinear, multiphasic nature of nanocarrier drug-release kinetics. On the other hand, CHS and TSA moved toward fewer deep networks or more aggressively tuned networks, which in turn, probably led to their lower predictive accuracy. The results of these experiments, which are corroborated by the cross-validation performance, TOPSIS ranking, and nonparametric statistical tests (Friedman and Kruskal–Wallis), are a collective affirmation that OOA is the most reliable and effective hyperparameter optimization tool for the present modeling framework and the best strategy for capturing complex physicochemical-kinetic relationships in drug-release systems.

**Table 3:** Optimized hyperparameters and their corresponding convergence iteration counts, offering insights into the tuning process and overall optimization efficiency.

Models	Optimizers	Convergence of 200 iterations (RMSE)	Optimized hyperparameters	
DNN	Osprey Optimization Algorithm	Min = 1.011 Mean = 1.350 Max = 2.344	Number of neurons per layer	128,128,64
			Learning Rate	0.01872
			Batch Size	32
			Epochs	120
	Crocodiles hunting strategy optimization	Min = 1.276 Mean = 1.806 Max = 3.030	Number of neurons per layer	96,48,24
			Learning Rate	0.087
			Batch Size	48
			Epochs	80
	Transient search algorithm	Min = 1.404 Mean = 1.814 Max = 2.962	Number of neurons per layer	192.96,48
			Learning Rate	0.0026
			Batch Size	64
			Epochs	80

### 4.2 Comprehensive multi-metric evaluation of optimized DNN variants using accuracy, efficiency, and robustness indicators

Table 4 articulates multi-metric model performance and discloses a distinct, cross-metric ranking of the present dataset and modeling setup: DNOA (Osprey-optimized hybrid) ≻ DNCO (Crocodile Hunting Strategy) ≻ DNTA (Transient Search) ≻ baseline DNN. DNOA has realized simultaneous improvements across accuracy (RMSE, MSE), explanatory power (R^2^), and robustness indexes (NSE, GR125) in both training and testing phases, thus, generalization beyond simple overfitting is implied. The decrease in test RMSE (from the baseline DNN to DNOA) that is accompanied by high R^2^ and NSE values consistently indicates that optimizer-guided hyperparameter tuning effectively reduces the training-testing gap and makes the predictions more stable. TOPSIS ranking and nonparametric tests (Friedman and Kruskal–Wallis) also noticed these differences as being statistically significant (*p* < 0.05) and thus they are not due to random variations. From the biological point of view, this multi-metric superiority is indicative of the enhanced ability of the model to capture both rapid burst release and slower sustained phases typical for nanocarrier kinetics, which means that complex physicochemical interactions are better modeled.

**Table 4:** Essential statistical metrics for assessing model performance, emphasizing predictive accuracy and overall efficiency.

Process	Category	ML Models	Evaluation metrics				
			RMSE	R^2^	MSE	NSE	GR125
Train	Single model	DNN	2.015	0.939	4.059	0.885	0.415
	Hybrid models	DNOA	0.917	0.988	0.841	0.976	0.736
		DNCO	1.163	0.974	1.354	0.962	0.708
		DNTA	1.370	0.957	1.876	0.947	0.481
Test	Single model	DNN	2.539	0.916	6.448	0.777	0.519
	Hybrid models	DNOA	1.324	0.974	1.760	0.939	0.704
		DNCO	1.642	0.957	2.705	0.906	0.556
		DNTA	1.527	0.937	2.348	0.919	0.630

### 4.3 Integrated Taylor diagram assessment of correlation, variability reproduction, and structural error among predictive models

Figure 4 employs a Taylor diagram to visually summarize an integrated assessment of model fidelity by layering correlation, standard deviation, and centered RMSD into one single visual framework. This method is especially effective for the nonlinear drug-release systems, where both the similarity of the pattern and the reproduction of the variability are necessary to capture the multiphasic kinetics correctly. By putting these metrics side by side, the diagram allows a thorough scrutiny of how closely each model resembles the structural features of the experimental release data. Their relative performances are made quite transparent. The OOA-optimized hybrid (DNOA) carries the least distance from the reference point; thus, it is indicative of almost perfect correlation (≈0.99) as well as good agreement in variance with minimal structural error. The CHS-based model (DNCO) is also a strong performer; however, its alignment with DNOA is a little less. The TSA-enhanced model (DNTA) shows moderate similarity, as it is better than the baseline but also has noticeable deviations in both correlation and RMSD. The unoptimized DNN, as anticipated, is at the largest distance from the reference; thus, it indicates that this model has a very limited capability in capturing nonlinear release behavior without metaheuristic refinement. Importantly, the Taylor diagram is an instrument that supports the main innovation of this work: metaheuristic-assisted DNN frameworks lead to a significant structural prediction accuracy increase. The clearly better variance reproduction and the notably lowered RMS error, especially in DNOA, are the factors that most clearly show the mechanistic pertinence of the optimized models for representing realistic diffusion-erosion dynamics in nanocarrier drug release.

**Figure 4:**
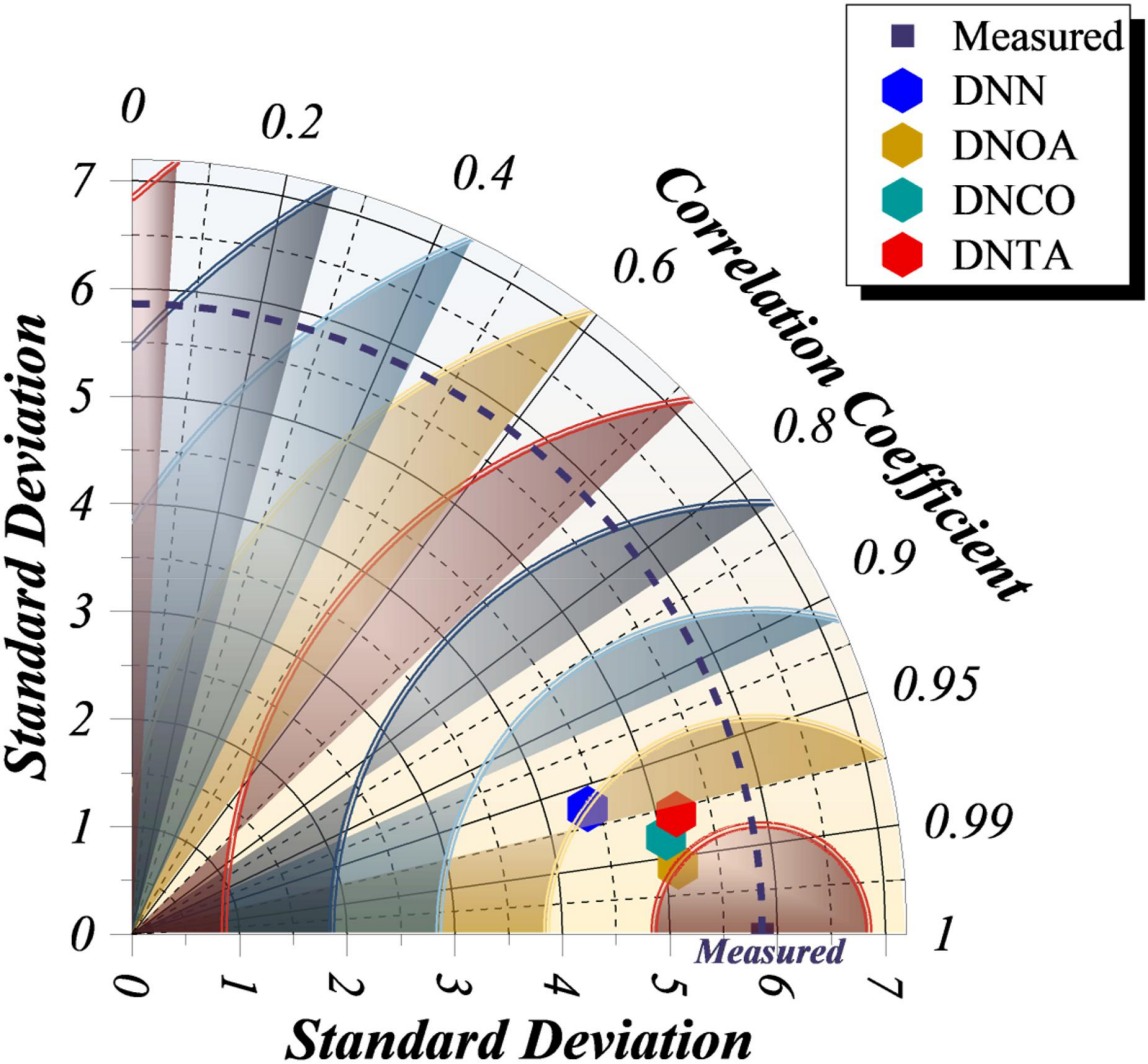
Taylor diagram for comparing the correlation and standard deviation of the models against the observed data, providing an overall assessment of model performance.

### 4.4 Residual analysis highlighting error compactness and generalization gains from metaheuristic optimization

Figure 5 explores the residuals for the baseline DNN and its optimizer-enhanced variants by looking at the way the prediction errors for training and testing samples are distributed. The baseline DNN reveals a wide and uneven error profile, which means that it has a high variance and that it frequently goes to the extreme regions of under- and over-prediction, which are, among other things, typical signs of weaker generalization. All optimized models, on the other hand, show to a great extent more compact and symmetric error distributions, thus reflecting their improved stability and tighter control of the residual variability. In particular, the Osprey-optimized model (DNOA) has the most beneficial pattern of errors: the errors are very close to zero with minimal high-error tails, and this closeness is ensured from training to testing; therefore, the model is strongly robust. DNCO is still quite well-balanced but has more moderate outliers, while DNTA has slightly wider dispersion. The overall journey is a confirmation that metaheuristic optimization is instrumental in reducing the residual spread, variance, and train–test consistency. Drug-release modeling, where nonlinear kinetics are involved, and models have to be capable of minimizing deviation across the full error spectrum in order to be used reliably for formulation design, is an example where such stabilization is of great importance.

**Figure 5:**
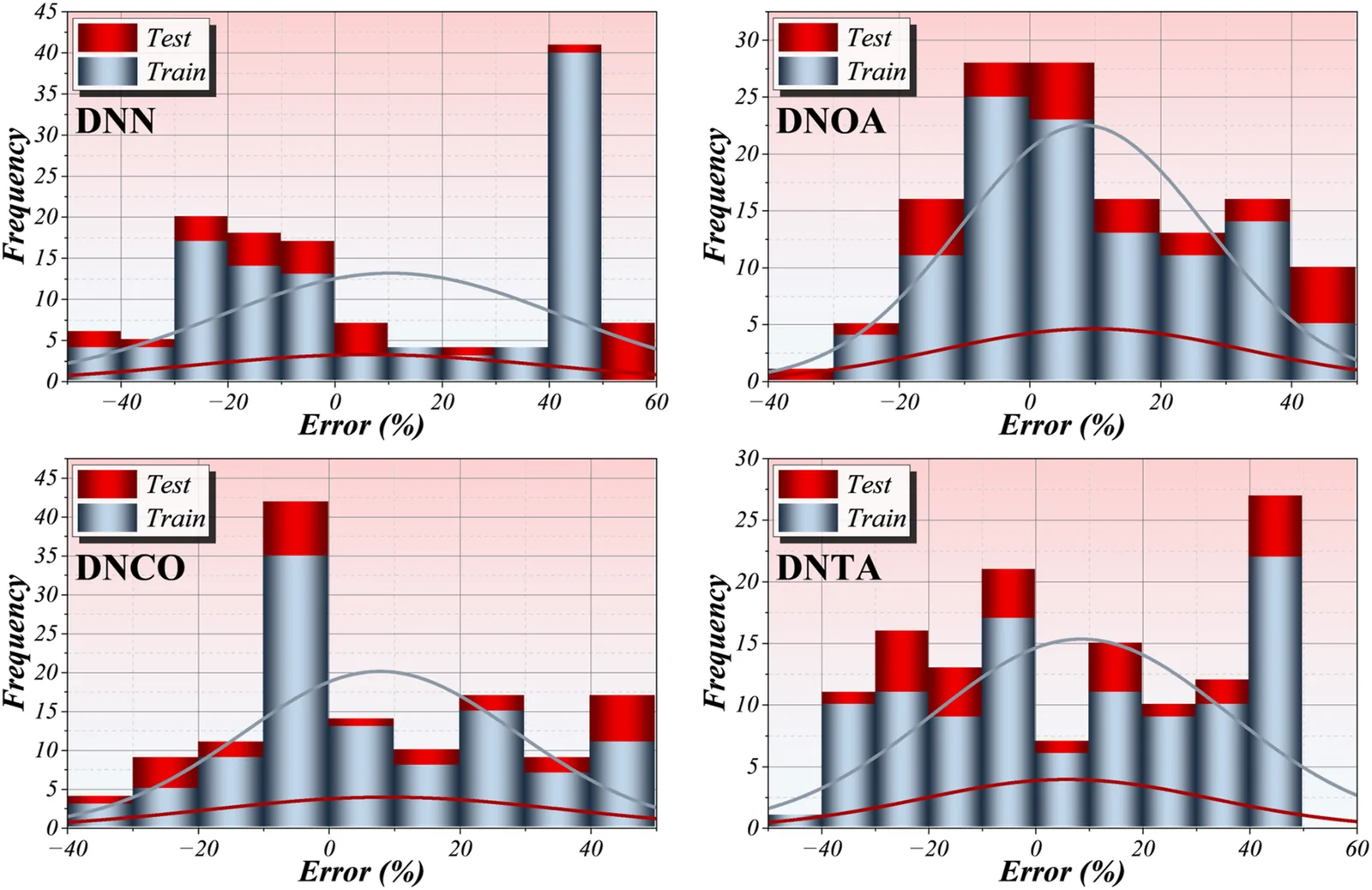
Stacked histogram for visualizing the distribution of prediction errors across different models or categories, providing an overall view of error patterns.

### 4.5 Three-dimensional multi-objective performance mapping of predictive models via R^2^, RMSE, and TOPSIS score integration

Figure 6 uses a 3D R^2^-RMSE-TOPSIS plot to summarize the performance of the predictive models in terms of multiple objectives. Such a single-figure representation is especially relevant for drug-release modeling, as a single accuracy metric may hide significant structural differences in the quality of predictions. By combining accuracy (R^2^), error magnitude (RMSE), and distance to the ideal solution (TOPSIS score), the figure makes it possible to order models in a balanced and fair manner, a necessary step for nonlinear, multiphasic release behavior analysis. The modeling pipeline clearly exhibits a performance gradient from one state to another. The baseline DNN without optimization shows the weakest overall profile and is the farthest from the ideal region. The stepwise improvement is the result of introducing metaheuristic tuning: the TSA-based model (DNTA) attains a better accuracy-error balance, and then the CHS-optimized hybrid (DNCO) is followed by demonstrating stronger structural consistency. The progression reaches its climax with the OOA-optimized model (DNOA), which is the closest to the ideal point and, therefore, accounts for the most advantageous trade-off of all the criteria evaluated.

**Figure 6:**
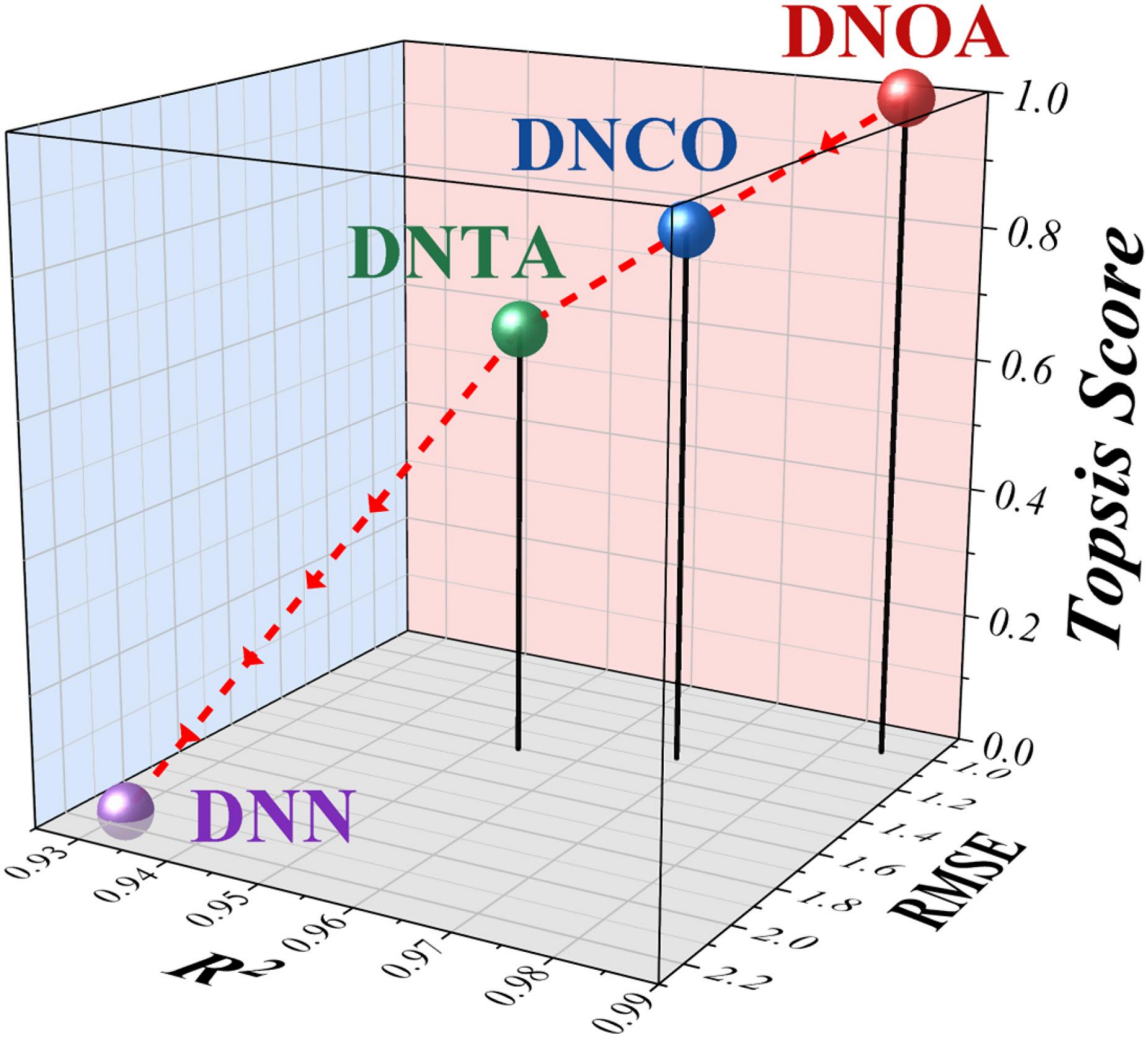
3D line-symbol plot for visualizing the TOPSIS scores of different models or options, highlighting their relative performance.

The integration of R^2^, RMSE, and TOPSIS scores provides a consolidated view of model performance by simultaneously considering predictive accuracy, error magnitude, and overall ranking consistency. Rather than serving as an additional measure of predictive capability, the TOPSIS analysis functions as a decision-support tool that facilitates comparison among competing models when multiple evaluation criteria are considered simultaneously. The resulting rankings are therefore interpreted in conjunction with the conventional performance metrics rather than as independent evidence of model superiority.

### 4.6 Non-parametric pairwise ranking of predictive models using the Friedman test heatmap for statistical performance differentiation

Figure 7 shows the heatmap obtained from the Friedman test, which is a distribution-free, rank-based comparison of the four predictive models that were evaluated on the same datasets. The use of the Friedman test is crucial in this case, as performance distributions in drug-release modeling are frequently non-normal due to nonlinear and multiphasic release behavior. By using a non-parametric framework, the analysis is free from erroneous assumptions and ensures that model comparisons indicate statistically significant differences rather than differences in variance or scale. Moreover, the heatmap modality helps to understand the pairwise rank relationships, details that are beyond the reach of standard metrics such as RMSE or R^2^. The findings showcase an unequivocal and easily understandable hierarchy of the models' performances. The unoptimized DNN and the TSA-enhanced DNTA are, in most cases, the lowest-ranked regions; thus, they are the weakest and statistically unfavorable under non-parametric evaluation. DNCO is in the middle zone, showing some, but not very significant, improvement. Contrarily, the OOA-optimized hybrid (DNOA) is the one that most of the time is found in the upper-ranked cells farthest from the diagonal, and it demonstrates repeatedly higher median ranks. The concentration of positive rankings around DNOA confirms that, within the Friedman framework, it is statistically superior, not just numerically better. Most importantly, Figure 6 serves as a statistical endorsement of the optimization pipeline. The clear separations between models signify that the enhancements due to OOA-based tuning are not random but, rather, systematic and reproducible.

**Figure 7:**
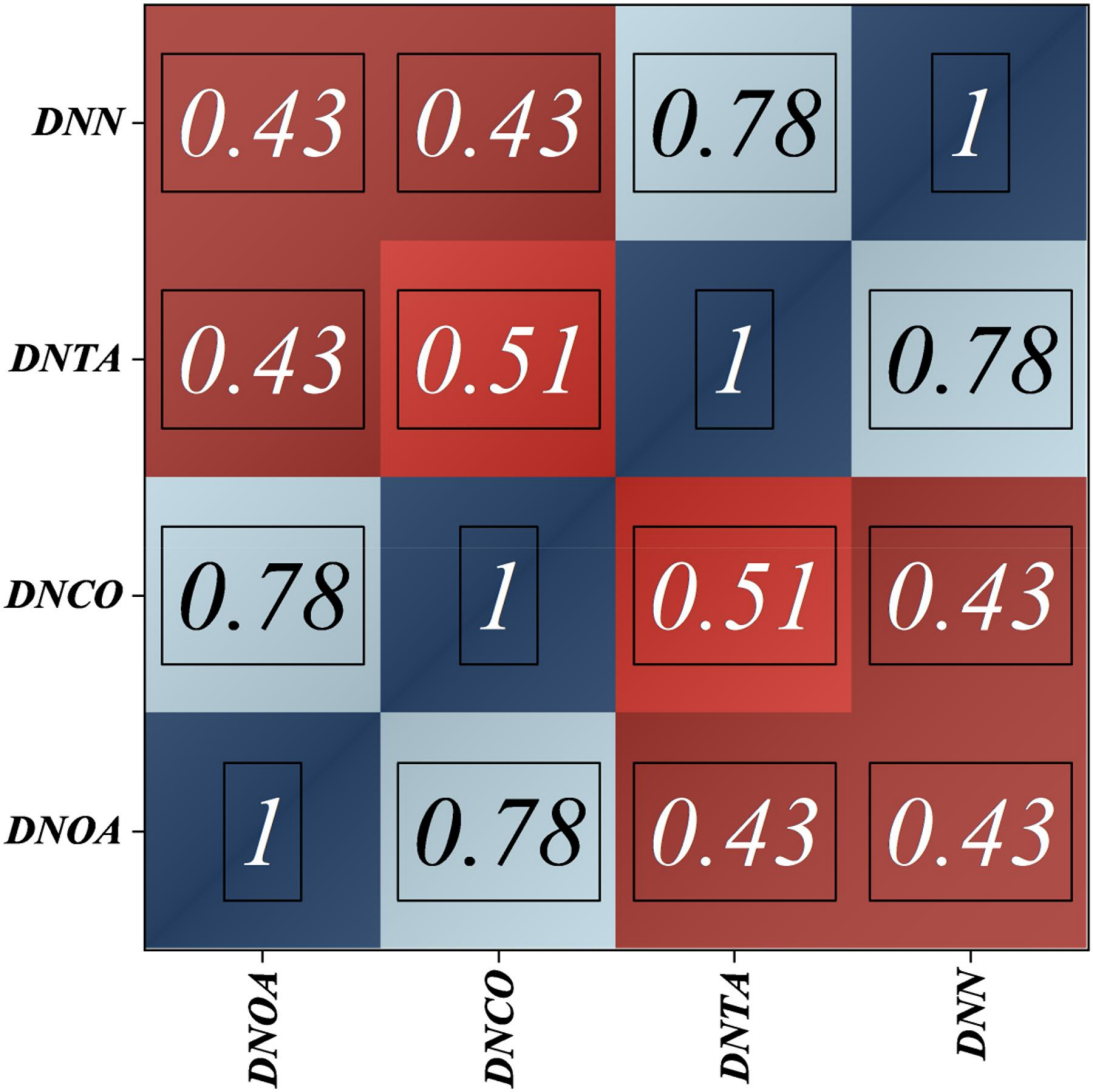
Heatmap plot for visualizing the results of the Friedman test, showing the distribution and differences among models.

The Friedman test was employed as a non-parametric statistical procedure to evaluate whether the relative rankings of the predictive models remained consistent across multiple evaluation settings. Its role is complementary to the performance metrics and TOPSIS analysis, providing statistical support for the observed ranking patterns without relying on distributional assumptions. Consequently, the Friedman analysis should be viewed as a validation of ranking consistency rather than a standalone indicator of model superiority. The results indicate that the relative ordering of the evaluated models remains stable across the considered performance criteria.

Figure 8 illustrates the SHAP-based feature impact analysis for the input variables using a parallel coordinate plot, showing how each feature contributes to the model's predictions across all samples. As shown in Figure 8, the model output is primarily driven by a subset of key descriptors, particularly Weight, Position, Size, Zeta potential, and DrugCarrierRatio, which exhibit strong variations in SHAP values and indicate high influence on prediction behavior. These features capture the most important nonlinear and interaction effects within the model. In contrast, logP, Solubility, Pgp, Comp1, and Comp2 show relatively moderate and dispersed contributions, suggesting that their effects are more dependent on interactions with other variables rather than acting as dominant predictors. The Route feature has the weakest and most stable influence, indicating a limited role in shaping the model output.

**Figure 8:**
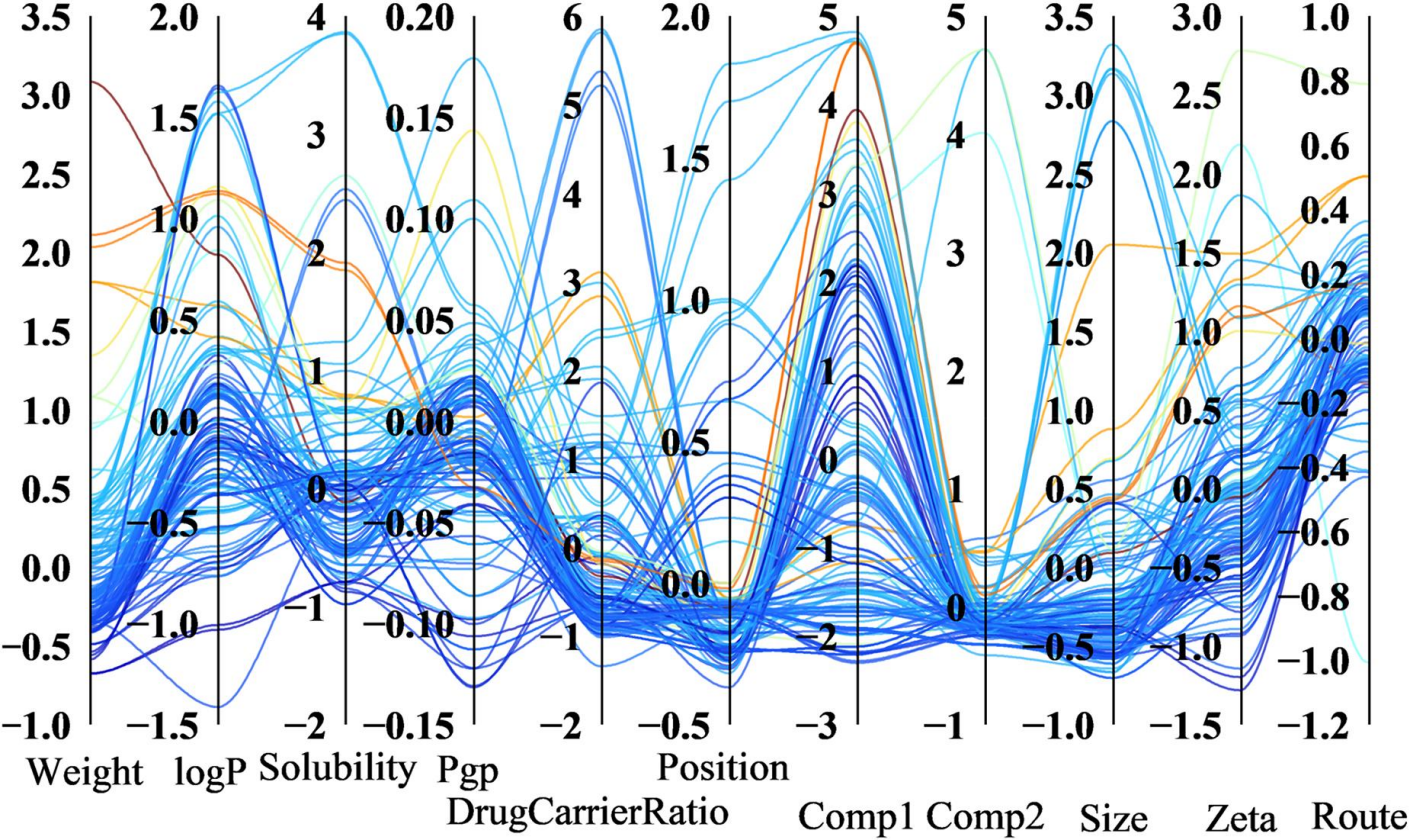
Feature analyses for the impact of the inputs based on SHAP.

Figure 9 presents the LIME-based local interpretability analysis of the model, showing feature-wise contributions across individual samples for all input variables. As illustrated in Figure 9, the LIME explanations reveal strong sample-dependent variability in feature influence, indicating that the model relies on different descriptors for different predictions rather than a single global pattern. Among the features, Weight, logP, Position, Comp1, and Size exhibit relatively large fluctuations across samples, suggesting that these variables have the most significant local impact on the model's decision-making process. In contrast, Pgp, DrugCarrierRatio, Comp2, Zeta, and Route show comparatively smaller and more stable contributions, indicating weaker or more context-dependent effects on individual predictions. The Route feature, in particular, demonstrates consistently low influence across samples, confirming its limited role in local prediction adjustments.

**Figure 9:**
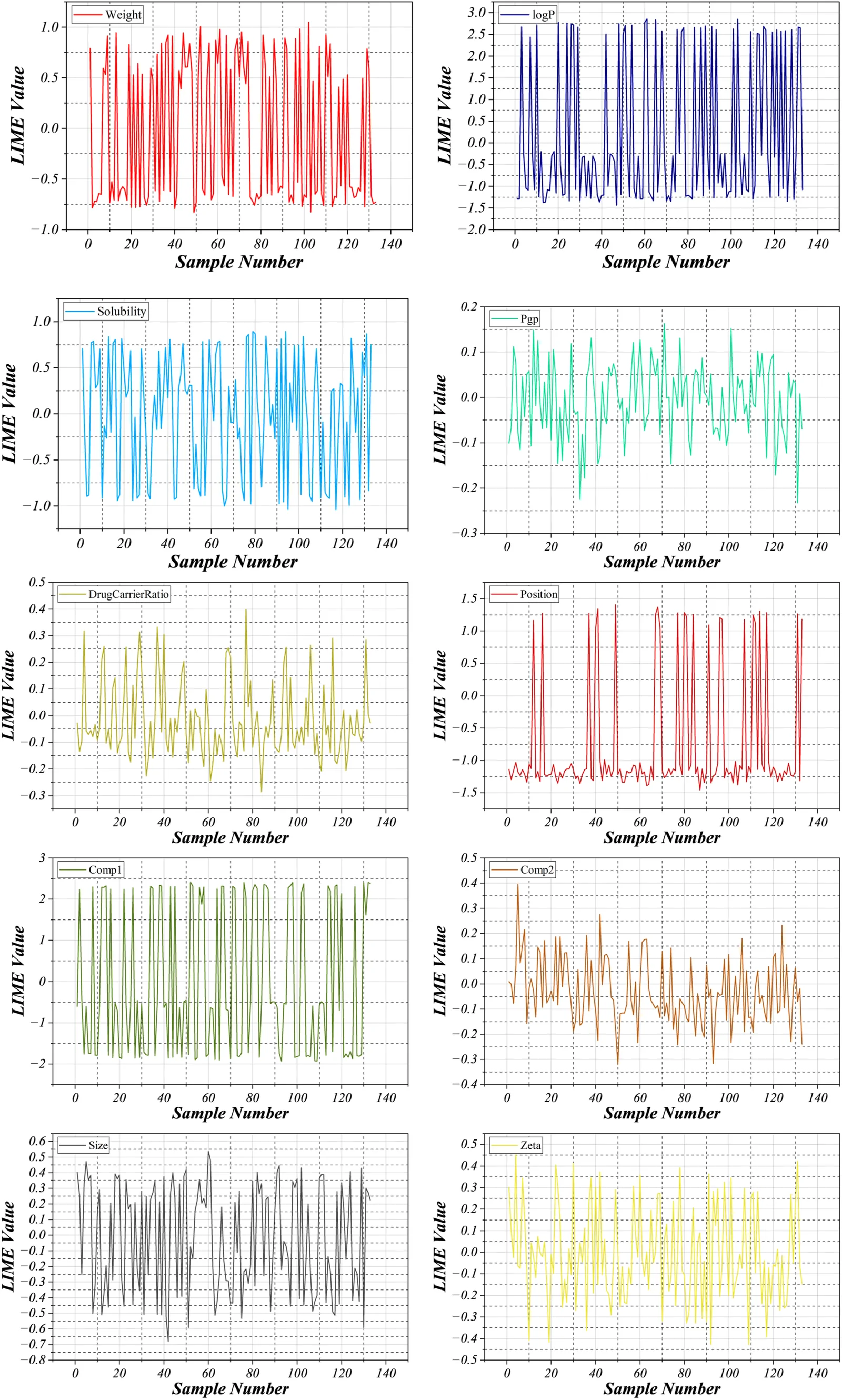
Feature analyses for the impact of the inputs based on LIME.

### 4.7 Interpretation of findings and translational significance

The evidence from this research reveals that the drug-release phenomenon in nanocarrier systems is nonlinear, heterogeneous, and influenced by multifactor interactions due to their intrinsic nature. The very strong skewness, heavy-tailed distributions, and the lack of linear clustering in the data indicate that the release dynamics cannot be structurally represented by classical linear or monotonic models. These features explain the change in methods to deep nonlinear architectures that can learn irregular, high-order dependencies without making any distributional assumptions. Feature analysis based on two criteria also indicates that complexity is beyond this: formulation descriptors such as drug–carrier ratio and zeta potential are places of high dispersion and considerable Kruskal–Wallis differentiation, thus releasing the main engines which operate through non-parametric, non-monotonic mechanisms rather than simple proportional relationships. The transition from the baseline DNN to the metaheuristic-enhanced variants thus illustrates a consistent hierarchy of modeling capability against this background. The main network of the baseline managed to capture the vital nonlinear structure; however, the bio-inspired optimizers (TSA, CHS, and, in particular, the OOA) are the ones that bring about the systematic refinement of the solution space. It is the OOA, in fact, that most of the time reflects superior convergence behavior, less error variance, and a more accurate matching of the experimental release profiles, thereby indicating a strong compatibility between the search dynamics it undertakes and the drug-release modeling problem's topology. OOA gains in accuracy, stability, and practical reliability as per the multi-metric evaluation framework that includes RMSE, R^2^, MSE, NSE, GR125, and TOPSIS. Other instruments, like Taylor diagrams and the Friedman test, are on the same page as they confirm that these advantages are statistically significant rather than random fluctuations.

These methodological innovations lead to a significant impact in the real world. The hybrid DNN-optimizer models that allow for early in-silico screening of formulation variables thus very much lessen the dependence on empirical trial-and-error, save expensive materials, and speed up formulation development pipelines, major industrial nanocarrier manufacturing goals. The industrial and regulatory considerations further the integration of multi-criteria evaluators and non-parametric statistical validation to bring the framework in line with QbD, MIDD, and digital formulation platforms that are at an early stage of development. Although retraining may be needed when the model is extended to new material classes or unusual release mechanisms, the performance and generalizability that have been shown give a feasible route to the acceleration of nanocarrier R&D and the enhancement of data-driven decision-making throughout the pharmaceutical development lifecycle. Moreover, the capacity to model diffusion-degradation interplay, burst release, and multiphasic kinetics paves the way for the rational design of sustained-, controlled-, and CNS-targeted delivery systems, thus enabling the therapeutic performance to be enhanced, the toxicity to be lowered, and the patient adherence to be increased.

### 4.8 Scope clarification and modeling rationale

It is important to clarify that the present study is focused on *in vitro* drug release behavior from nanocarrier systems, modeled using formulation- and carrier-related physicochemical descriptors extracted from heterogeneous literature sources. The developed framework is designed to capture the relationship between nanocarrier characteristics and experimentally observed release profiles under controlled conditions, rather than to represent *in vivo* pharmacokinetic processes or systemic disposition mechanisms directly.

It is acknowledged that *in vivo* drug performance is influenced by multiple physiological factors, including plasma protein binding, tissue distribution, clearance mechanisms, and the free–bound drug fraction (e.g., Kpuu), particularly in central nervous system applications. However, such parameters are not consistently available across reported *in vitro* release studies and therefore cannot be incorporated into the present modeling framework.

In addition, physicochemical properties of the drug, such as logP, logD, and pKa, may significantly influence absorption and distribution behavior. Nevertheless, the present analysis prioritizes formulation- and carrier-specific descriptors, including particle size, surface charge, morphology, encapsulation efficiency, drug localization, and release conditions, as these variables are more consistently reported and directly govern release kinetics within nanocarrier systems.

Furthermore, physiologically based pharmacokinetic (PBPK) and mechanistic modeling approaches, including frameworks such as PBNB, provide established methodologies for estimating *in vivo* drug release and disposition. The present study does not aim to replace such mechanistic approaches but instead provides a complementary data-driven predictive framework for *in vitro* release modeling, which may be integrated with mechanistic pharmacokinetic models in future multi-scale investigations.

## 5 Conclusion

Drug-release behavior in nanoparticle carriers is non-linear, multiphasic, and influenced by a variety of factors that interact in a heterogeneous manner. Traditional kinetic or mechanistic models cannot sufficiently capture these factors. The treatment of skewed, heavy-tailed distributions and significant non-parametric differentiation among descriptors, such as drug-carrier ratio, zeta potential, particle size, and structural characteristics, points to the intricate, multi-dimensional aspects of these systems. Combining DNN with bio-inspired metaheuristic optimizers (TSA, CHS, and OOA) in a hybrid predictive framework reveals gradual improvements in predictive performance. The OOA-optimized DNN was able to reach the lowest minimum RMSE (1.011), the lowest mean RMSE (1.350), and the highest R^2^ (∼0.99), reflecting not only superior convergence stability but also a closer match to the experimental release profiles. Validation using multiple metrics such as NSE, GR125, RMSE, R^2^, and TOPSIS indicates that the improvements are consistent in terms of accuracy, generalizability, and structural fidelity. In addition, Friedman and Kruskal–Wallis tests offer non-parametric statistical significance (*p* < 0.05) evidence. These findings are of great pragmatic value for pharmaceutical formulations and nanocarrier materials. Predicting in-silico with high accuracy the interplay of diffusion–degradation, burst release, and matrix-controlled kinetics can lessen the need for trial-and-error experimentation, thereby saving materials and speeding up the development process. Moreover, dependable modeling is a key factor in the transition from the laboratory to the pilot or industrial scale, where a slight change in particle size, surface charge, or lipid composition may lead to a significant change in release behavior. From the clinical point of view, it paves the way for the rational design of sustained- or targeted-release therapies such as CNS-oriented formulations, thus dosing frequency, patient compliance, and safety can be improved. Although the performance of the model is influenced by the diversity of the dataset and might need a retraining of the model for new materials or an unusual release mechanism, the results are consistent with the establishment of a strong and scalable framework. Subsequent research can take this approach to cover more types of nanoparticles, study the long-term stability and biodegradation rate, and also collect *in vivo* pharmacokinetic and pharmacodynamic data to increase prediction accuracy. These developments would lead to a greater application of the framework in the design of nanocarriers guided by models and facilitate a wider transition into clinical and industrial sectors. The optimization analysis performed in this study is limited to a comparison among selected nature-inspired metaheuristic algorithms (TSO, CHS, and OOA). Although these approaches demonstrated effective hyperparameter tuning capabilities within the investigated framework, standard optimization methods such as grid search, random search, and Bayesian optimization were not included as benchmark strategies. Consequently, the reported results should be interpreted as a comparative assessment among the selected metaheuristic optimizers rather than evidence of superiority over all available hyperparameter optimization techniques. Future work may incorporate both conventional and advanced optimization approaches to enable a broader evaluation of optimization performance in drug release prediction tasks.

## Data Availability

The original contributions presented in the study are included in the article/Supplementary Material, further inquiries can be directed to the corresponding author.
